# Definition of Local Recurrence Site in Resected Pancreatic Adenocarcinoma: A Multicenter Study (DOLORES-1)

**DOI:** 10.3390/cancers13123051

**Published:** 2021-06-18

**Authors:** Alessandra Arcelli, Federica Bertini, Silvia Strolin, Gabriella Macchia, Francesco Deodato, Savino Cilla, Salvatore Parisi, Aldo Sainato, Michele Fiore, Pietro Gabriele, Domenico Genovesi, Francesco Cellini, Alessandra Guido, Silvia Cammelli, Milly Buwenge, Emiliano Loi, Silvia Bisello, Matteo Renzulli, Rita Golfieri, Alessio G. Morganti, Lidia Strigari

**Affiliations:** 1 Radiation Oncology, IRCCS Azienda Ospedaliero-Universitaria di Bologna, 40138 Bologna, Italy; federica.bertini@studio.unibo.it (F.B.); alessandra.guido@aosp.bo.it (A.G.); silvia.cammelli2@unibo.it (S.C.); milly.buwenge2@unibo.it (M.B.); silvia.bisello@studio.unibo.it (S.B.); alessio.morganti2@unibo.it (A.G.M.); 2 Department of Experimental, Diagnostic and Specialty Medicine–DIMES, Alma Mater Studiorum, Bologna University, 40138 Bologna, Italy; rita.golfieri@unibo.it; 3 Medical Physics, IRCCS Azienda Ospedaliero-Universitaria di Bologna, 40138 Bologna, Italy; silvia.strolin@aosp.bo.it (S.S.); emiliano.loi@irst.emr.it (E.L.); lidia.strigari@aosp.bo.it (L.S.); 4 Radiation Oncology Unit, Gemelli Molise Hospital, Università Cattolica del Sacro Cuore, 86100 Campobasso, Italy; gabriella.macchia@unicatt.it (G.M.); francesco.deodato@unicatt.it (F.D.); 5 Istituto di Radiologia, Università Cattolica del Sacro Cuore, 00168 Roma, Italy; francesco.cellini@policlinicogemelli.it; 6 Medical Physics Unit, Gemelli Molise Hospital, Università Cattolica del Sacro Cuore, 86100 Campobasso, Italy; savino.cilla@unicatt.it; 7 Unit of Radiation Therapy, IRCCS Casa Sollievo della Sofferenza, 71013 San Giovanni Rotondo, Italy; s.parisi@operapadrepio.it; 8 Radiation Oncology, Pisa University Hospital, 56126 Pisa, Italy; a.sainato@ao-pisa.toscana.it; 9 Radiation Oncology, Campus Bio-Medico University, 00128 Rome, Italy; m.fiore@unicampus.it; 10 Radiation Therapy, Candiolo Cancer Institute–FPO, IRCCS Candiolo, 10060 Candiolo, Italy; pietro.gabriele@ircc.it; 11 Department of Radiation Oncology, SS. Annunziata Hospital, G. D’Annunzio University of Chieti, 66100 Chieti, Italy; d.genovesi@unich.it; 12 Fondazione Policlinico Universitario A. Gemelli, IRCCS, UOC di Radioterapia, Dipartimento di Scienze Radiologiche, Radioterapiche ed Ematologiche, 00168 Roma, Italy; 13 Radiology Unit, IRCCS Azienda Ospedaliero-Universitaria di Bologna, 40138 Bologna, Italy; matteo.renzulli@aosp.bo.it

**Keywords:** pancreatic neoplasms, adjuvant chemoradiation, pattern of failure, Kernel density estimation

## Abstract

**Simple Summary:**

Pancreatic cancer remains a disease with a dismal outlook for patients, with high relapse rates after surgery and adjuvant treatments. Thanks to the high conformality achievable with advanced radiotherapy techniques, a more robust definition of clinical target volume (CTV) margins is mandatory. Moreover, a precise CTV definition may affect local control, minimizing radiation-related toxicity and allowing dose escalation. Contrary to two recent studies, RTOG contouring guidelines are not based on a pattern of failure analysis. We provided a local failure risk map in resected pancreatic cancer, validating the results of previous studies. Moreover, according to a new probabilistic approach, we provided new CTV contouring guidelines for the postoperative radiotherapy of pancreatic cancer, modeling targets’ margins on a combination of our validated local failure map (30% of local failures) and RTOG guidelines (70% of local failures).

**Abstract:**

The study aimed to generate a local failure (LF) risk map in resected pancreatic cancer (PC) and validate the results of previous studies, proposing new guidelines for PC postoperative radiotherapy clinical target volume (CTV) delineation. Follow-up computer tomography (CT) of resected PC was retrospectively reviewed by two radiologists identifying LFs and plotting them on a representative patient CT scan. The percentages of LF points randomly extracted based on CTV following the RTOG guidelines and based on the LF database were 70% and 30%, respectively. According to the Kernel density estimation, an LF 3D distribution map was generated and compared with the results of previous studies using a Dice index. Among the 64 resected patients, 59.4% underwent adjuvant treatment. LFs closer to the root of the celiac axis (CA) or the superior mesenteric artery (SMA) were reported in 32.8% and 67.2% cases, respectively. The mean (± standard deviation) distances of LF points to CA and SMA were 21.5 ± 17.9 mm and 21.6 ± 12.1 mm, respectively. The Dice values comparing our iso-level risk maps corresponding to 80% and 90% of the LF probabilistic density and the CTVs-80 and CTVs-90 of previous publications were 0.45–0.53 and 0.58–0.60, respectively. According to the Kernel density approach, a validated LF map was proposed, modeling a new adjuvant CTV based on a PC pattern of failure.

## 1. Introduction

The five-year overall survival rate for pancreatic cancer (PC) is only 8% [1] and, by 2030, PC will be the second leading cause of cancer death in the US [2]. Even in the minority (20%) of PC patients with resectable tumors at diagnosis, the relapse rate after surgery and adjuvant treatments is still high [3,4,5]. Some autopsy series of patients who underwent pancreatectomy reported 75–83% and 75% local-regional disease relapse and systemic failure rates, respectively [6,7]. However, advancements in systemic therapies, both in the adjuvant and neoadjuvant settings, could lead to a reduced incidence of distant metastases. Consequently, local-regional recurrences may become more of an issue. PC local failure (LF) is generally unrelated to the clinical stage at the initial presentation [8] and is difficult to detect using radiological imaging [9].

Based on several randomized trials, the adjuvant treatment of resected PC is based on chemotherapy (CHT) [10,11,12] or concurrent chemoradiation (CRT) [13,14,15,16]. However, the addition of CRT to adjuvant CHT is still controversial due to the findings of the ESPAC-1 randomized trial showing a detrimental effect of CRT [11]. Nevertheless, several limitations of that trial should be considered: (i) treatment fields were not included in the protocol, (ii) the radiotherapy (RT) planning was not subjected to central review, and (iii) the prescribed dose was particularly low (40 Gy delivered with a split-course regimen) [17].

Conversely, several pieces of evidence suggest a clear impact of the CRT total dose and quality on a patient’s outcome. In fact, a secondary analysis of the Radiation Therapy Oncology Group (RTOG) 9704 randomized trial [18] showed a significant correlation between adherence to protocol quality assurance (QA) guidelines and outcome in terms of local control and overall survival. Moreover, a pooled analysis of 955 patients with resected PC showed the significant impact of the institutional volume in terms of radiation treatments per year (< 10 versus ≥ 10) on survival in this setting. The authors concluded that these results confirm the impact of RT quality on a patient’s prognosis in PC adjuvant treatment. [19] Finally, two pooled analyses reported a significant correlation among total dose and survival in PC patients treated with postoperative CRT [20,21].

The use of modern RT techniques, such as volumetric modulated arc therapy (VMAT), could favor an increasingly precise and faster irradiation of the target, thus allowing a reduced irradiation of the organs at risk (OARs) and a lower risk of intra-fraction variations.

Obviously, the delivery of a higher radiation dose in resected PC requires an accurate target definition to improve the probability of local control and reduce the risk of side effects in the surrounding organs at risk. In this regard, stepwise contouring guidelines for the definition of the target in this setting were published in 2012 by the RTOG [22]. However, those guidelines were proposed by a consensus committee of six radiation oncologists with expertise in gastrointestinal RT without a pattern of failure analysis.

More recently, two series reported LF risk maps in resected PC patients [23,24]. Those maps were generated based on the spatial site of LF in the tumor bed and regional nodes to define a postoperative target volume, including higher-risk areas.

Based on this background, the first aim of this analysis was to generate a local failure risk map in resected pancreatic cancer, validating the results of the latter studies [23,24]. The second objective was to propose new guidelines for PC adjuvant target delineation based on Kernel density estimation [25] according to a finite data sample representing a three-dimensional (3D) LF distribution map.

## 2. Materials and Methods

### 2.1. Study Design

This is a retrospective, multicenter, observational study. On behalf of AIRO (Italian Association of Radiation and Clinical Oncology), we collected data and imaging (contrast-enhanced computer tomography (CT)) of resected PC patients with LF from six Italian centers.

### 2.2. Endpoints

The primary aim of the study was to validate the results of two studies [23,24], proposing the clinical target volume (CTV) for adjuvant RT, modeled to encompass 80% and 90% PC LF. The secondary purpose of this study was to generate a 3D LF map of the high-risk volume for PC recurrences, taking some anatomical landmarks as reference points. Moreover, our purpose was to propose new guidelines for CTV delineation generated through the Kernel density estimation [25].

### 2.3. Eligibility Criteria

We included in this analysis patients with pancreatic adenocarcinoma and LF detected on CT scans after pancreaticoduodenectomy.

### 2.4. Three-Dimensional Local Recurrence Map

Two expert radiologists (MR, RG), each with more than 15 years of experience in gastrointestinal tumors, performed the segmentation of LF volume on follow-up contrast-enhanced CT images using Pinnacle (Philips Healthcare, Fitchburg, WI, USA). After identifying the axial CT slices corresponding to the LF cranial and caudal levels, the central geometric level of the LF was defined as the central slice between the latter. In this central slice, the relapse was inscribed in a rectangle whose geometric center was considered as the geometric center of the relapse. Thereafter, we measured the distance between the LF center and some anatomical landmarks, including the root of the celiac axis (CA), the root of the superior mesenteric artery (SMA), and the medial edge of the pancreatic stump in the anterior-posterior, cranio-caudal, and latero-lateral directions, respectively.

Finally, a map of relapses was generated by plotting the LF centers on the contrast-enhanced CT of a representative patient, without anatomical variations, who had previously undergone pancreaticoduodenectomy. LFs were displayed by drawing a 5 mm-diameter sphere around the LF center on the reference patient’s CT using the Pinnacle software.

### 2.5. Contouring

**Figure 1:**
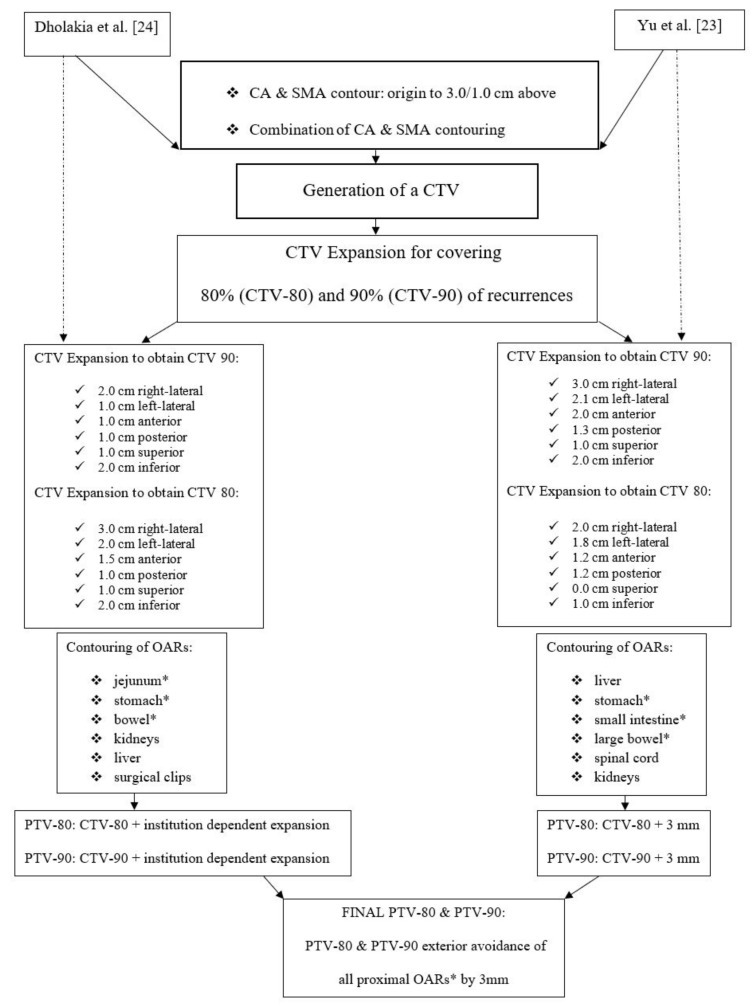
Stepwise planning process based on Dholakia et al. [24] and Yu et al. [23].

On the representative patient’s CT, both CA and SMA were contoured from the slide corresponding to their origin up to the slides 1 and 3 cm below, respectively. Then, the CA and SMA contours were merged to define a single CTV. The latter was expanded following the instructions proposed by Yu et al. [23] and Dholakia et al. [24]. Both papers provided guidelines to generate a CTV-80 potentially encompassing 80% of LF and a CTV-90 potentially encompassing 90% of LF. Subsequently, two PTVs-80 and two PTVs-90 were defined based on the same studies’ maps. Details on the contouring process are provided in Figure 1. Moreover, the standard postoperative CTV and PTV for resected PC, based on the RTOG contouring consensus guidelines [22], were drawn on the representative patient CT.

### 2.6. Statistical Analysis

CT images, contours, and points of interest of the representative patient were imported from Pinnacle into MIM version 5.2 (MIM Software Inc., Cleveland, OH, USA) and transferred to a MATLAB workspace (Release 2019 a, The MathWorks, Inc., Natick, MA, USA) using an MIM extension (i.e., using an ad hoc script written in Java) to convert the DICOM contours and coordinates in a mask and DICOM coordinates of LF in elements of noxel space $\left\{x_{n,i},y_{n,i}\right\}$.

Random recurrence points $\left\{x_{\mathrm{CTVs},i},y_{\mathrm{CTVs},i}\right\}$ were generated using the CTV based on the RTOG guidelines for PC patients undergoing postoperative RT (named standard CTV contour—i.e., CTVs). To calculate the 3D LF risk map, we randomly extracted 70% of the random recurrence points $\left\{x_{\mathrm{CTVs},i},y_{\mathrm{CTVs},i}\right\}$ from the RTOG CTVs to be combined with 30% of the centroids randomly obtained from the registered LF points $\left\{x_{n,i},y_{n,i}\right\}$, according to previous studies [8,9].

Using this approach, an 3D LF risk map can be calculated using a weighted overlay of LF areas $\left\{x_{i},y_{i}\right\},$ I = 1,..., N obtained from both RTOG CTVs and experimental data.

All LF centroids were imported into the “Kernel Density” tool to calculate the probabilistic density per slice. Kernel density estimation is a non-parametric method used to estimate the probability density function of a random variable. Kernel density estimation is a fundamental data smoothing problem where inference is based on a finite data sample. Using a MATLAB script, the bivariate Kernel function was calculated as follows:

$$\hat{f}\left(x,y,j\right)=\frac{1}{N_{j}h_{x}h_{y}}\sum_{i=1}^{N_{j}}K\left(\;\frac{x_{i}-x}{h_{x}},\frac{y_{i}-y}{h_{y}}\right),$$

where $h_{x}$ and $h_{y}$ are the smoothing coefficients, $\left\{x_{i},y_{i}\right\}$, and I = 1, ...,$\;N_{j}$ are the recurrence points in slice j containing $N_{j}$ recurrence points. This function transforms the “sharp” point location into a circular area around each $\left\{x_{i},y_{i}\right\}$ coordinate slice by slice to obtain the radial Kernel estimator based on the Euclidian distance as follows:

$$\hat{f}\left(x,y\right)=\frac{1}{Nh_{x}h_{y}}\sum_{i=1}^{N}K\left(\sqrt{{\left(\;\frac{x_{n,i}-x}{h_{x}}\right)}^{2}+{\left(\frac{y_{n,i}-y}{h_{y}}\right)}^{2}}\right),$$

where the distance between recurrence points and the CA/SMA centroid were also calculated on the reference CT images. Finally, the resulting calculation map was saved in a matrix and reported as a DICOM dose within MIM and Pinnacle to visualize the 3D LF maps and extract the iso-levels.

### 2.7. Comparison with the Results of Other Studies

The CTV-90 and the CTV-80, encompassing 90% and 80% of the plotted recurrences, respectively, calculated as iso-level contours from the 3D probabilistic map, were compared with the corresponding volumes calculated by Yu et al. [23] and Dholakia et al. [24] using the Dice and Jaccard indexes [26] within MIM Vista (MIM Vista Software, version 5.2). The Dice and Jaccard coefficients are similar statistical measures of the spatial overlap between two volumes as follows:

Dice(A,B) = 2 |A∩B||A|+|B|,
Jaccard(A,B) = |A∩B||A∪B|,

A and B denotes the volumes to be compared. Here, Dice is defined as 2 × intersection volume/total sum of volumes, while Jaccard describes the volume of the intersection between two volumes/volume of the union of these volumes. Both overlap coefficients normalize the degree of intersection from 0 (no overlap) to 1 (perfect overlap).

In addition, based on the mean distance from CA and SMA for each subgroup reported in the papers by Yu et al. [23] and Dholakia et al. [24], the weighted distances from SMA and CA have been calculated and compared with our results.

### 2.8. Ethical Issues

All enrolled patients had to sign a written informed consent. The study (DOLORES: Definition Of LOcal REcurrence Sites) was approved by our institutional review board (202/2015/O/Oss).

## 3. Results

Sixty-four patients were included in this analysis. Patients’ characteristics are shown in Table 1. Most patients (59.4%) underwent adjuvant treatment.

**Figure 2:**
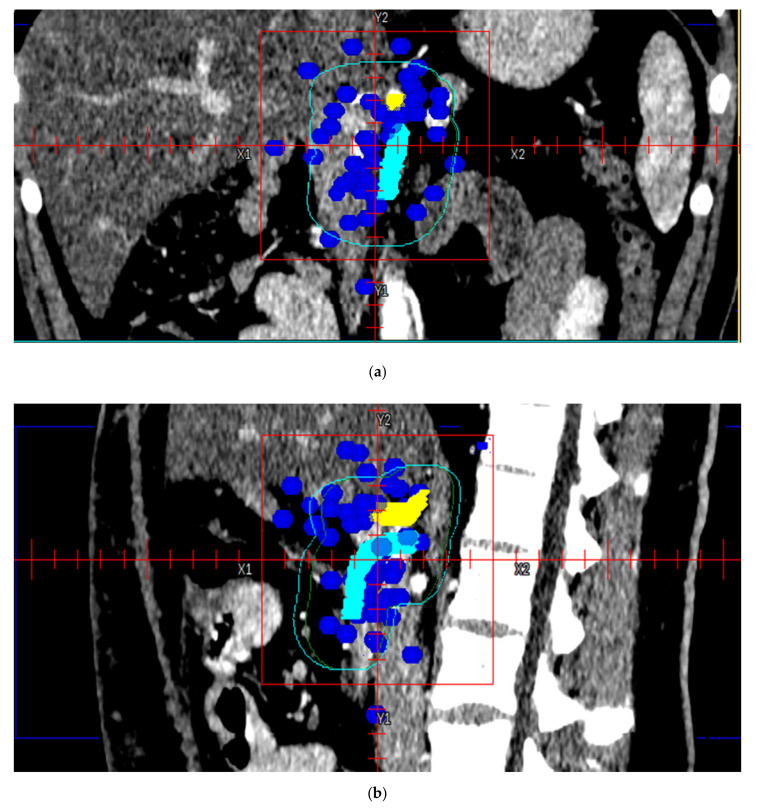
Local recurrence sites (blue symbols) plotted with respect to the pancreatic stump, CA (yellow) and SMA (cyan). The light blue line and green line represent CTV 90 Yu and CTV 90 Dholakia, respectively. Coronal (**a**) and sagittal view (**b**).

The LF map plotted with respect to the pancreatic stump, CA, and SMA is shown in Figure 2a,b.

**Figure 3:**
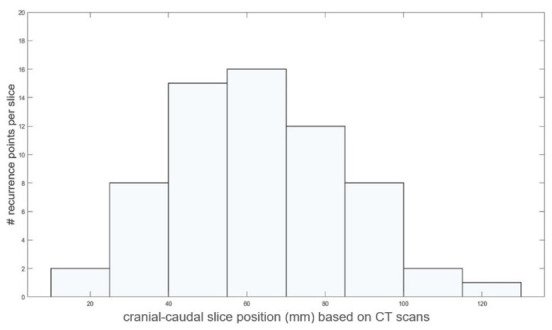
A histogram of the distribution number of recurrence points according to their position along the cranio-caudal direction (in mm) reported on the representative patient shown in Figure 2.

The number of LF points according to the position along the cranial-caudal direction is shown in Figure 3. Twenty-one (32.8%) patients experienced LF closer to the CA, with a mean and standard deviation of the distance from the CA of 21.5 mm and 17.9 mm, respectively, while 43 patients (67.2%) experienced LF closer to the SMA, with a mean and standard deviation of the distance from the SMA of 21.6 mm and 12.1 mm, respectively.

In our cohort, the mean ± standard deviation distances of LF points from CA and SMA were 21.5 ± 17.9 mm and 21.6 ± 12.1 mm, respectively, which were larger than the findings of Dholakia et al. (16 ± 10 mm and 6.4 ± 5 mm, respectively) and similar to those reported by Yu et al. (19.7 ± 22 mm and 16.2 ± 18.5 mm, respectively).

The similar coefficients between CTVs (CTV80 and CTV90) calculated by Dholakia et al. and Yu et al. and the corresponding CTVs extracted as the iso-level of the LF probability in this analysis are shown in Table 2. The Dice values were around 0.45 for CTV80s and 0.58 for CTV90s, while the Jaccard values were also lower.

**Figure 4:**
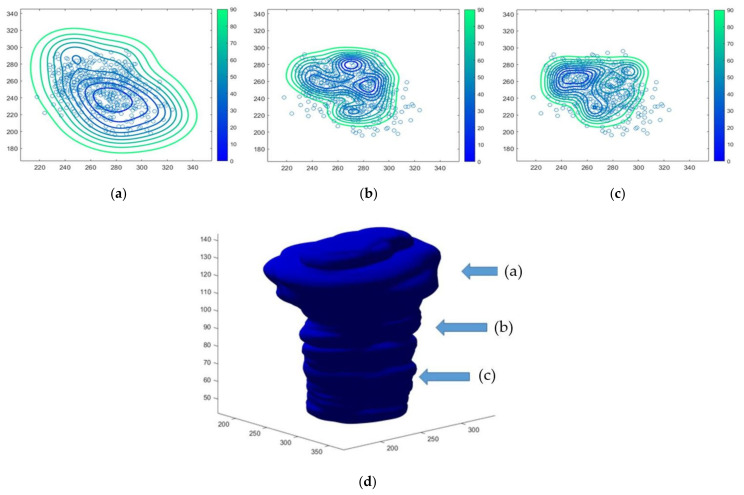
Three representative examples (**a**,**b**,**c**) of the iso-level local failures probabilistic density in the noxel space at the height are indicated by the corresponding raw indicated in (**d**). Open symbols represent the recurrence points randomly generated using RTOG 0848 CTV and experimental recurrence database. The 3D CTV80 corresponds to an 80% iso-level risk (**d**).

Figure 4a–c show the spatial distribution of the calculated probabilistic density using the “Kernel Density” tool in three representative slices of noxel space indicated by the raw a, b, and c in Figure 4d. The 3D iso-map risk level corresponding to 80% of the LF probability is also shown in Figure 4d.

**Figure 5:**
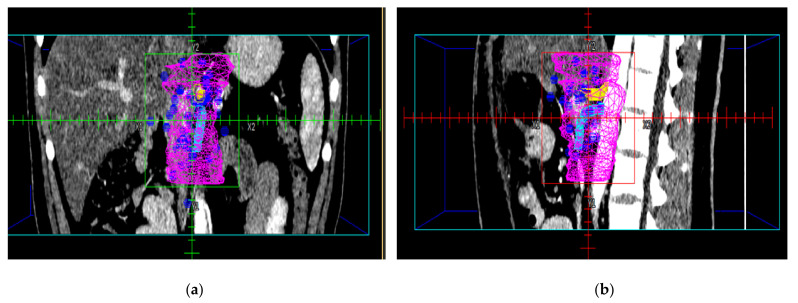
Iso-level recurrence maps were transferred to MIM and Pinnacle, as shown in Figure 4. Coronal (**a**) and sagittal view (**b**).

The LF map was imported in MIM and Pinnacle as a 3D dose file to generate iso-level maps and perform a comparison with the other delineated volumes (Figure 5).

The minimal distances from CA and SMA of CTV80 and CTV90 extracted as the iso-level of the LF probability are reported in Table 3.

**Figure 6:**
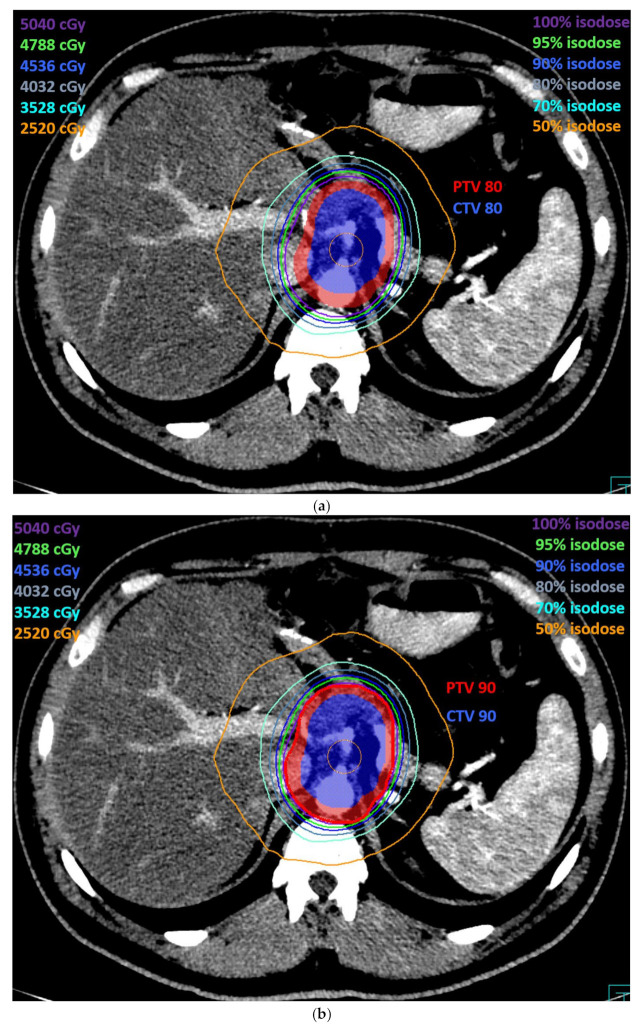
The axial view of a VMAT plan dosimetry covering the new proposed region of interest with regard to CTV80, PTV80 (**a**), CTV90, and PTV 90 (**b**).

Figure 6 shows the dose distribution in an axial scan for the irradiation of CTV80 (Figure 6a) and CTV90 (Figure 6b), respectively, defined according to the method described in this report and planned by VMAT.

The relative planning target volumes were obtained by adding 10 mm to the cranio-caudal direction and 5 mm to the lateral-lateral and anterior-posterior direction.

## 4. Discussion

The risk of LF represents a significant problem in PC patients undergoing surgical resection [6,7]. In subjects undergoing postoperative RT, given the need for adequate doses [20,21], the possibility to accurately define the LF pattern would be very useful. Notably, the irradiation of smaller CTVs would allow the delivery of high RT doses by reducing the risk of side effects.

The CTV represents the volume of tissue containing a demonstrable GTV and/or a subclinical disease with a clinically relevant probability of occurrence (i.e., higher than 5–10%). CTV delineation is generally based on clinical experience or consensus among expert radiation oncologists.

Autoptic studies show that tumor cells can invade areas beyond the CTV, thus potentially increasing the LF risk. Standard-of-care RT plans uniformly irradiate the CTV, including the visible tumor, and are extended by a non-uniform margin. However, the extent of this margin can vary even by a few centimeters across different institutions.

To calculate novel margins and validate the proposed ones, a statistical/mathematic approach has been applied. Our study demonstrates that the calculation of the risk map involving the estimates of probability density functions can be effectively accomplished using the Kernel density methods. Kernel density estimation has been widely studied and a univariate implementation is readily available in MATLAB to be adapted for the specific context. The radiographic site of LF in reference to major abdominal vessels allows the use of vascular anatomy as a landmark to build a standard and reproducible target that includes areas at high risk for LF. In the identification of quantitative margins, it would be advantageous to adopt a probabilistic CTV definition based on the expected number of clonogenic cells according to the ICRU 83 definition [27], thus allowing a further reduction in target volumes/margins and consequently favoring dose escalation, decreased treatment-related toxicity, or a combination of the these. Of note, our approach incorporates both the prior knowledge based on RTOG 0848 CTV and the experimental LF information in a methodology computationally tractable to determine an empirical probability distribution.

Our choice to generate the risk map using a combination of both the RTOG guidelines and our validated local failure map seems justified by the results of a recent study. Patel et al. [28] analyzed the patterns of LF after adjuvant stereotactic RT in PCs resected with a close or positive surgical margin. In their experience, the CTV was delineated by omitting elective nodal irradiation and, in some cases, excluding the abutting vasculature. Forty-two per cent of patients experienced LF during the follow-up and most of the latter (60.7%) were outside the PTV, while the large majority of them (92.9%) would have been encompassed by the RTOG volume. The authors concluded that target expansion should be considered in future studies on hypofractionated adjuvant RT in this setting [28].

To our knowledge, this represents the first study appointing the probabilistic Kernel density approach to extract an LF map. Notably, as shown in Table 2 the Dice and Jaccard indexes are similar for the volumes obtained comparing CTV/PTV 80/90 from Yu et al. [23] and Dholakia et al. [24] to those of this work. Nevertheless, some variations among the different results by Yu et al. [23], Dholakia et al. [24], and our report emerged. These differences could be attributed to random differences in LF distribution, the different methodology adopted for margin extraction, and the anatomical variations resulting from the probable inhomogeneity of ethnic origin and BMI among representative patients of the reported analyses.

Indeed, our CTVs, and, consequently, the expansions’ CTV margins we proposed (Table 3), are larger than the ones provided by Yu et al. [23], Dholakia et al. [24], and the RTOG consensus guidelines [22]. Moreover, our CTV-80 and CTV-90 are modeled on a map including the pancreatic, nodal, and retro-pancreatic failures of patients receiving adjuvant treatment with RT and/or CHT, or not. Conversely, Yu et al. [23] considered for their map only cases with recurrences not receiving adjuvant RT, localized only around the resection area or the peri-pancreatic vessels or the retroperitoneal nodes. Moreover, Dholakia et al. [24] did not point out whether the local failures included in their map were pancreatic or nodal. Nevertheless, the CTV margins we provided almost overlap with the RTOG consensus volumes, except that they are 1 cm longer in the caudal direction. This caudal larger margin seems to be supported by findings of the aforementioned report [28]. Patel et al. [28] reported that 7.1% of LFs are not covered by RTOG consensus volumes, localized exactly outside the caudal extent of the recommended RTOG CTV.

Our results encourage the application of this methodology on larger patient cohorts with PC LFs. The redefined CTVs/PTVs could result in improved local tumor control, but their validation in a prospective trial is necessary.

Future clinical trials are needed to verify the feasibility and effectiveness of the proposed target definition. Actually, an observational validation study on adjuvant chemoradiation, planned according to the target definition described in this report, is underway in our centers.

This analysis reports an optimization method for the definition of the target in the setting of PC postoperative chemoradiation. However, we should admit that interest in neoadjuvant therapies, even in resectable PC, is progressively growing [29]. However, up-front surgical resection is unlikely to be completely removed from the available treatment options for resectable PC. Therefore, we believe that this method may also be useful in the future.

Our report has some limits due to the retrospective design of the study. Firstly, for our analysis we selected only patients after pancreaticoduodenectomy from the DOLORES database. Due to the scarcity of patients treated with total pancreatectomy, a separate analysis was not performed. Consequently, our results can only be applied to patients who underwent pancreaticoduodenectomy. This could have impacted the different spatial distributions of LF points observed by Yu et al. [23], who also included LF after total pancreatectomy in their analysis.

## 5. Conclusions

Thanks to the high conformality achievable with advanced treatment planning systems and the high performance and accurate delivery of novel accelerators [30], a more robust definition of CTV margins is mandatory. 

In fact, a precise CTV definition may affect local control minimizing radiation-related toxicity and allow dose escalation. According to both the PC pattern of failure and the RTOG guidelines, a new postoperative CTV was proposed and modeled on our validated LF map. The adoption of the Kernel density approach was feasible and automatic in our study and might represent an effective tool for a probabilistic PC postoperative CTV definition.

## Figures and Tables

**Table 1:** Patients’ characteristics.

Variable	Value	Patients (%)
Age (years)	Median (range)	66.5 (37–83)
Gender	Male	43 (67.2)
	Female	21 (32.8)
Grading	1	8 (12.5)
	2	37 (57.8)
	3	19 (29.7)
Tumor site	Head	51 (79.7)
	Body	13 (20.3)
Tumor diameter (cm)	Median (range)	2.6 (0.9–5.2)
*p*T	2	13 (20.3)
	3	44 (68.7)
	4	7 (11.0)
*p*N	0	26 (40.6)
	1	38 (59.4)
Number of pathological positive nodes	Median (range)	3 (0–40)
Number of resected nodes	Median (range)	23 (3–60)
Site of the local recurrences	Pancreatic	24 (37.5)
	Retropancreatic	14 (21.9)
	Nodal	26 (40.6)
Type of surgery	Pancreatico-duodenectomy	45 (70.3)
	pylorus preserving pancreatico-duodenectomy	19 (29.7)
Surgical margins	R0	34 (53.1)
	R1	30 (46.9)
Interval surgery–relapse (months)	Median (range)	10 (3–47)
Adjuvant therapy	Chemotherapy	17 (26.6)
	Chemoradiation	3 (4.7)
	Chemotherapy + chemoradiation	18 (28.1)
	Missing	26 (40.6)

**Table 2:** Similar coefficients between CTV80 and CTV90 calculated by Yu et al. [23] and Dholakia et al. [24], respectively, and the corresponding CTVs extracted as iso-level recurrence probability in the present series.

Contour 1	Contour 2	Dice	Jaccard
CTV80 [present series]	CTV80 [Dholakia]	0.53	0.36
CTV80 [present series]	CTV80 [Yu]	0.45	0.29
CTV80 [Dholakia]	CTV80 [Yu]	0.42	0.27
CTV90 [present series]	CTV90 [Dholakia]	0.58	0.41
CTV90 [present series]	CTV90 [Yu]	0.60	0.43
CTV90 [Dholakia]	CTV90 [Yu]	0.57	0.40

**Table 3:** Minimal distance from the celiac axis and the superior mesenteric artery of CTV80 and CTV90 extracted from the iso-level recurrence probability at 80% and 90%, respectively.

Directions	Clinical Target Volume (CTV)	Minimal Distance from Vessels of CTV (cm)	
		Celiac Axis (CA)	Superior Mesenteric Artery (SMA)
right-lateral	CTV80	1.5	2.0
	CTV90	1.8	2.3
left-lateral	CTV80	1.6	1.2
	CTV90	1.7	1.2
anterior	CTV80	1.7	0.9
	CTV90	1.8	1.5
posterior	CTV80	0.7	2.0
	CTV90	1.0	2.5
superior	CTV80	1.5	2.0
	CTV90	1.5	2.0
inferior	CTV80	0.0	2.0
	CTV90	0.5	2.4

## Data Availability

The data presented in this study are available upon request from the corresponding author.

## References

[1] Siegel R.L., Miller K.D., Jemal A. (2019). Cancer statistics, 2019. CA Cancer J. Clin..

[2] Rahib L., Smith B.D., Aizenberg R., Rosenzweig A.B., Fleshman J.M., Matrisian L.M. (2014). Projecting cancer incidence and deaths to 2030: The unexpected burden of thyroid, liver, and pancreas cancers in the United States. Cancer Res..

[3] Smeenk H.G., van Eijck C.H., Hop W.C., Erdmann J., Tran K.C., Debois M., van Cutsem E., van Dekken H., Klinkenbijl J.H., Jeekel J. (2007). Long-term survival and metastatic pattern of pancreatic and periampullary cancer after adjuvant chemoradiation or observation: Long-term results of EORTC trial 40891. Ann. Surg..

[4] Griffin J.F., Smalley S.R., Jewell W., Paradelo J.C., Reymond R.D., Hassanein R.E., Evans R.G. (1990). Patterns of failure after curative resection of pancreatic carcinoma. Cancer.

[5] Tepper J., Nardi G., Sutt H. (1976). Carcinoma of the pancreas: Review of MGH experience from 1963 to 1973. Analysis of surgical failure and implications for radiation therapy. Cancer.

[6] Hishinuma S., Ogata Y., Tomikawa M., Ozawa I., Hirabayashi K., Igarashi S. (2006). Patterns of recurrence after curative resection of pancreatic cancer, based on autopsy findings. J. Gastrointest. Surg..

[7] Kayahara M., Nagakawa T., Ohta T., Kitagawa H., Ueno K., Tajima H., Elnemr A., Miwa K. (1999). Analysis of paraaortic lymph node involvement in pancreatic carcinoma: A significant indication for surgery?. Cancer.

[8] Iacobuzio-Donahue C.A., Fu B., Yachida S., Luo M., Abe H., Henderson C.M., Vilardell F., Wang Z., Keller J.W., Banerjee P. (2009). DPC4 gene status of the primary carcinoma correlates with patterns of failure in patients with pancreatic cancer. J. Clin. Oncol..

[9] Tanaka H., Takamori H., Kanemitsu K., Chikamoto A., Beppu T., Baba H. (2012). An autopsy study to clarify characteristics of local recurrence after extended pancreatectomy with intraoperative radiation therapy in patients with pancreatic cancer. Langenbecks Arch. Surg.

[10] Neoptolemos J.P., Dunn J.A., Stocken D.D., Almond J., Link K., Beger H., Bassi C., Falconi M., Pederzoli P., Dervenis C. (2001). Adjuvant chemoradiotherapy and chemotherapy in resectable pancreatic cancer: A randomised controlled trial. Lancet.

[11] Neoptolemos J.P., Stocken D.D., Friess H., Bassi C., Dunn J.A., Hickey H., Beger H., Fernandez-Cruz L., Dervenis C., Lacaine F. (2004). A randomized trial of chemoradiotherapy and chemotherapy after resection of pancreatic cancer. N. Engl. J. Med..

[12] Oettle H., Post S., Neuhaus P., Gellert K., Langrehr J., Ridwelski K., Schramm H., Fahlke J., Zuelke C., Burkart C. (2007). Adjuvant chemotherapy with gemcitabine vs observation in patients undergoing curative-intent resection of pancreatic cancer: A randomized controlled trial. JAMA.

[13] Kalser M.H., Ellenberg S.S. (1985). Pancreatic cancer. Adjuvant combined radiation and chemotherapy following curative resection. Arch. Surg..

[14] Gastrointestinal Tumor Study Group (1987). Further evidence of effective adjuvant combined radiation and chemotherapy following curative resection of pancreatic cancer. Cancer.

[15] Klinkenbijl J.H., Jeekel J., Sahmoud T., van Pel R., Couvreur M.L., Veenhof C.H., Arnaud J.P., Gonzalez D.G., de Wit L.T., Hennipman A. (1999). Adjuvant radiotherapy and 5-fluorouracil after curative resection of cancer of the pancreas and periampullary region: Phase III trial of the EORTC gastrointestinal tract cancer cooperative group. Ann. Surg..

[16] Garofalo M.C., Regine W.F., Tan M.T. (2006). On statistical reanalysis, the EORTC trial is a positive trial for adjuvant chemoradiation in pancreatic cancer. Ann. Surg..

[17] Corsini M.M., Miller R.C., Haddock M.G., Donohue J.H., Farnell M.B., Nagorney D.M., Jatoi A., McWilliams R.R., Kim G.P., Bhatia S. (2008). Adjuvant radiotherapy and chemotherapy for pancreatic carcinoma: The Mayo Clinic experience (1975–2005). J. Clin. Oncol..

[18] Abrams R.A., Winter K.A., Regine W.F., Safran H., Hoffman J.P., Lustig R., Konski A.A., Benson A.B., Macdonald J.S., Rich T.A. (2012). Failure to adhere to protocol specified radiation therapy guidelines was associated with decreased survival in RTOG 9704—A phase III trial of adjuvant chemotherapy and chemoradiotherapy for patients with resected adenocarcinoma of the pancreas. Int. J. Radiat. Oncol. Biol. Phys..

[19] Morganti A.G., Falconi M., van Stiphout R.G., Mattiucci G.C., Alfieri S., Calvo F.A., Dubois J.B., Fastner G., Herman J.M., Maidment B.W. (2014). Multi-institutional pooled analysis on adjuvant chemoradiation in pancreatic cancer. Int. J. Radiat. Oncol. Biol. Phys..

[20] Morganti A.G., Cellini F., Buwenge M., Arcelli A., Alfieri S., Calvo F.A., Casadei R., Cilla S., Deodato F., Di Gioia G. (2019). Adjuvant chemoradiation in pancreatic cancer: Impact of radiotherapy dose on survival. BMC Cancer.

[21] Hall W.A., Colbert L.E., Liu Y., Gillespie T., Lipscomb J., Hardy C., Kooby D.A., Prabhu R.S., Kauh J., Landry J.C. (2013). The influence of adjuvant radiotherapy dose on overall survival in patients with resected pancreatic adenocarcinoma. Cancer.

[22] Goodman K.A., Regine W.F., Dawson L.A., Ben-Josef E., Haustermans K., Bosch W.R., Turian J., Abrams R.A. (2012). Radiation Therapy Oncology Group consensus panel guidelines for the delineation of the clinical target volume in the postoperative treatment of pancreatic head cancer. Int. J. Radiat. Oncol. Biol. Phys..

[23] Yu W., Hu W., Shui Y., Zhu X., Li C., Ren X., Bai X., Yu R., Shen L., Liang T. (2016). Pancreatic cancer adjuvant radiotherapy target volume design: Based on the postoperative local recurrence spatial location. Radiat. Oncol.

[24] Dholakia A.S., Kumar R., Raman S.P., Moore J.A., Ellsworth S., McNutt T., Laheru D.A., Jaffee E., Cameron J.L., Tran P.T. (2013). Mapping patterns of local recurrence after pancreaticoduodenectomy for pancreatic adenocarcinoma: A new approach to adjuvant radiation field design. Int. J. Radiat. Oncol. Biol. Phys..

[25] Davies T.M., Marshall J.C., Hazelton M.L. (2018). Tutorial on kernel estimation of continuous spatial and spatiotemporal relative risk. Stat. Med..

[26] Taha A.A., Hanbury A. (2015). Metrics for evaluating 3D medical image segmentation: Analysis, selection, and tool. BMC Med. Imaging.

[27] International Commission on Radiation Units and Measurements ICRU (2010). Prescribing, Recording and Reporting Photon Beam Therapy.

[28] Patel A.K., Rodríguez-López J.L., Bahary N., Zureikat A.H., Burton S.A., Heron D.E., Olson A.C. (2020). Patterns of failure after adjuvant stereotactic body radiation therapy for pancreatic cancer with close or positive margins. Adv. Radiat Oncol.

[29] Silvestris N., Longo V., Cellini F., Reni M., Bittoni A., Cataldo I., Partelli S., Falconi M., Scarpa A., Brunetti O. (2016). Neoadjuvant multimodal treatment of pancreatic ductal adenocarcinoma. Crit. Rev. Oncol. Hematol..

[30] Cellini F., Arcelli A., Simoni N., Caravatta L., Buwenge M., Calabrese A., Brunetti O., Genovesi D., Mazzarotto R., Deodato F. (2020). Basics and frontiers on pancreatic cancer for radiation oncology: Target delineation, SBRT, SIB technique, MRgRT, particle therapy, immunotherapy, and clinical guidelines. Cancers.

